# In Situ TEM Study of Size-Controlled Bi Quantum Dots in an Annealed GaAsBi/AlAs Multiple Quantum Well Structure

**DOI:** 10.1021/acsomega.4c10631

**Published:** 2025-03-06

**Authors:** Martynas Skapas, Esperanza Luna, Sandra Stanionytė, Karl Graser, Renata Butkutė

**Affiliations:** 1 Center for Physical Science and Technology, Saulėtekio av. 3, Vilnius LT-10257, Lithuania; 2 Paul Drude Institutu Solid State Electronics, Hausvogteiplatz 5−7, Berlin DE-10117, Germany

## Abstract

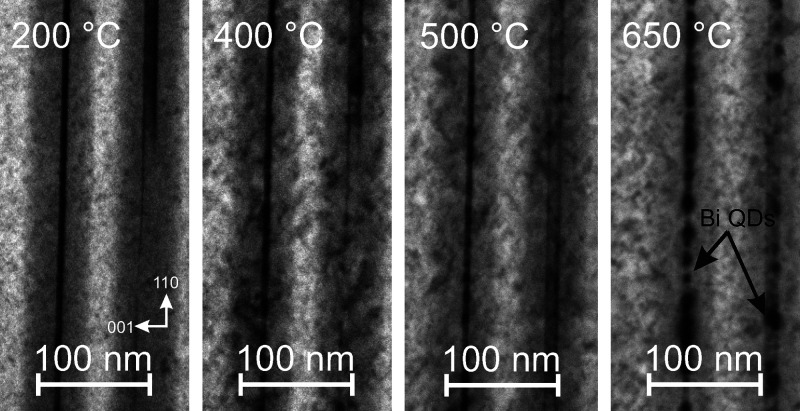

An in situ transmission electron microscopy study of Bi quantum dot (QD) formation in an annealed GaAsBi/AlAs multiple quantum well (MQW) structure is presented in this work. The investigated structure, containing two GaAsBi QWs and embedded in an AlGaAs parabolic quantum barrier (PQB), was grown on semi-insulating GaAs (100) and was transferred onto an in situ heating holder (DENS solutions) and heated up to 650 °C. Sample evolution was continuously recorded in situ in bright-field STEM mode. The analysis revealed that QD formation occurs at lower annealing temperatures in case of *in situ* heating of lamella than in bulk. In addition, we find that the mechanism governing Bi QD formation is different in the in situ TEM experiment compared to bulk ex-situ annealing. Comparison of the *ex-situ* and *in situ* annealed structures, as well as in-depth postannealed structure TEM analysis, is presented.

## Introduction

GaAsBi -based heterostructures have a large potential for optoelectronic applications in a wide spectral range extending from the near- to mid-infrared regions. In contrast to alloying by In or Sn,^1^ substitution of As by Bi in the GaAs lattice produces a much larger reduction in the band gap (−60 to −80 meV/%Bi), thus making this material attractive for infrared radiation emitters^2^ and detectors.^3^ New devices containing GaAsBi active layers are already reported—for instance, 1.06 μm-wavelength^2^ and 1.23 μm-wavelength^4^ GaAsBi/GaAs MQW-based light emitting diode with low Bi segregation; 1.142 μm-wavelength GaAsBi/GaAs single quantum well laser^5^ is also reported.

One of the outstanding challenges in the MBE growth of Bi-containing GaAs heterostructures is the low growth temperature (less than 450 °C) requirement for incorporation of Bi content above 5%, as this increases the density of nonradiative recombination centers. Postgrowth high-temperature (650 °C and above) annealing is a commonly employed procedure, which allows to improve the quality of the as-grown crystal and to reduce the density of nonradiative recombination defects. However, the effect of annealing on GaAsBi is still ambiguous. It has been shown by our group^6^ as well as by other researchers^7,8^ that the annealing at temperatures above 600 °C leads to the formation of Bi nanoparticles and the reduction in Bi content of the surrounding GaAsBi layer and onset of intense photoluminescence (PL) in the wavelength range from 1.35 to 1.5 μm. These Bi nanoparticles, provided their size is less than 20 nm, act as quantum dots, as Bi becomes a direct semiconductor with the decrease of QD size^6^ and by cathodoluminescence measurements.^9^

High-resolution transmission electron microscopy (HRTEM) studies provide very detailed information about the structure of the Bi nanoparticles (NPs) that are formed in Ga(As,Bi) after annealing. In particular, the formation of various Bi NPs of sizes from 7.6 to 22 nm in thick GaAsBi layers was reported.^7^ The authors show that, under specific conditions, the formed clusters were composed solely of a rh-Bi phase and that Bi QD (102) planes are nearly parallel to GaAs (220), although other authors reported that these rh-Bi nanoclusters are crystallographically incoherent with the GaAsBi matrix.^10^ Spontaneous creation of highly uniform arrays of Bi QDs inside the 3QW structure was also reported,^11^ indicating that Bi QD formation during MBE growth relies on a spinodal surface decomposition at early growth stages. The study of the 5xQW structure of alternating Bi-poor and Bi-rich GaAsBi layers grown by metalorganic vapor phase epitaxy (MOVPE) shows that 45 min annealing at 800 °C creates Bi QDs inside the more Bi-rich QWs. The Bi QD density is about 0.7–3.4 × 10^22^ m^–3^ with the higher density for the top QWs. Atomic probe tomography (APT) and HRTEM analysis showed a uniform Bi incorporation into GaAsBi layers under MOVPE growth conditions,^8^ although other studies reported nonuniform Bi distribution with chain-like ordering even before annealing, which is responsible for Bi NPs in the annealed samples.^12^ Formation of rh-Bi NPs was also reported in GaPBi.^13^ The authors reported that Bi nanoclusters orient in such a way that Bi (101) planes are parallel to GaPBi (202) with nonuniform size distribution of Bi QDs. Furthermore, GaAsBi layers may also be used as strain-compensation layers for high-quality InAs QD growth, because Bi also acts as a surfactant and provides more uniform size distribution of InAs QDs.^14−16^

There are no published works on *in situ* analysis of Bi quantum dot formation in the GaAsBi system during postgrowth annealing experiments. In our previous studies,^17−19^ formation of Bi QDs during *ex-situ* RTA annealing has been reported, and similar results have been published in refs (7,10,13,20). On the other hand, Bi NP formation decomposing various Bi-containing salts during *in situ* TEM annealing has been reported in refs (21−24). In this regime, Bi droplets undergo multiple melting–crystallization cycles.

In this work, we investigate the evolution of the microstructure of GaAsBi/AlGaAs MQW with parabolically graded barriers (PGBs), with special interest in checking the formation of nanostructures during *in situ* annealing up to 650 °C at the TEM.

## Results and Discussion

The GaAsBi/AlGaAs QW structures were grown using the Veeco GENxplor R&D molecular beam epitaxy MBE system equipped with standard cells for metallic Al, Ga, and Bi materials as well as arsenic cells containing independently controlled thermal zones for the bulk evaporator and the cracking head to generate pure As_2_ flux. The semi-insulating GaAs substrate oriented in the (100) crystalline plane was used for the deposition of QW structures. The substrate temperature was monitored by thermocouple readings with a precision of 1 °C.

Prior to the GaAsBi growth, the native oxide from the semi-insulating GaAs substrate was outgassed at 700 °C for 30 min and under maximum arsenic flux until a well-expressed (2 × 4) reflection high-energy electron diffraction (RHEED) pattern evidenced that native oxide was desorbed. An unintentionally doped 100 nm-thick GaAs buffer and 200 nm-thick AlGaAs barrier layers were deposited under the standard conditions of 665 °C substrate temperature and about 5 × 10^–6^ Torr As_2_ flux in order to provide a flat surface for epitaxial growth. After this procedure, the RHEED pattern usually showed a strong 2 × 4 reconstruction, which indicates the smooth surface and layer-by-layer growth.

**Figure 1:**
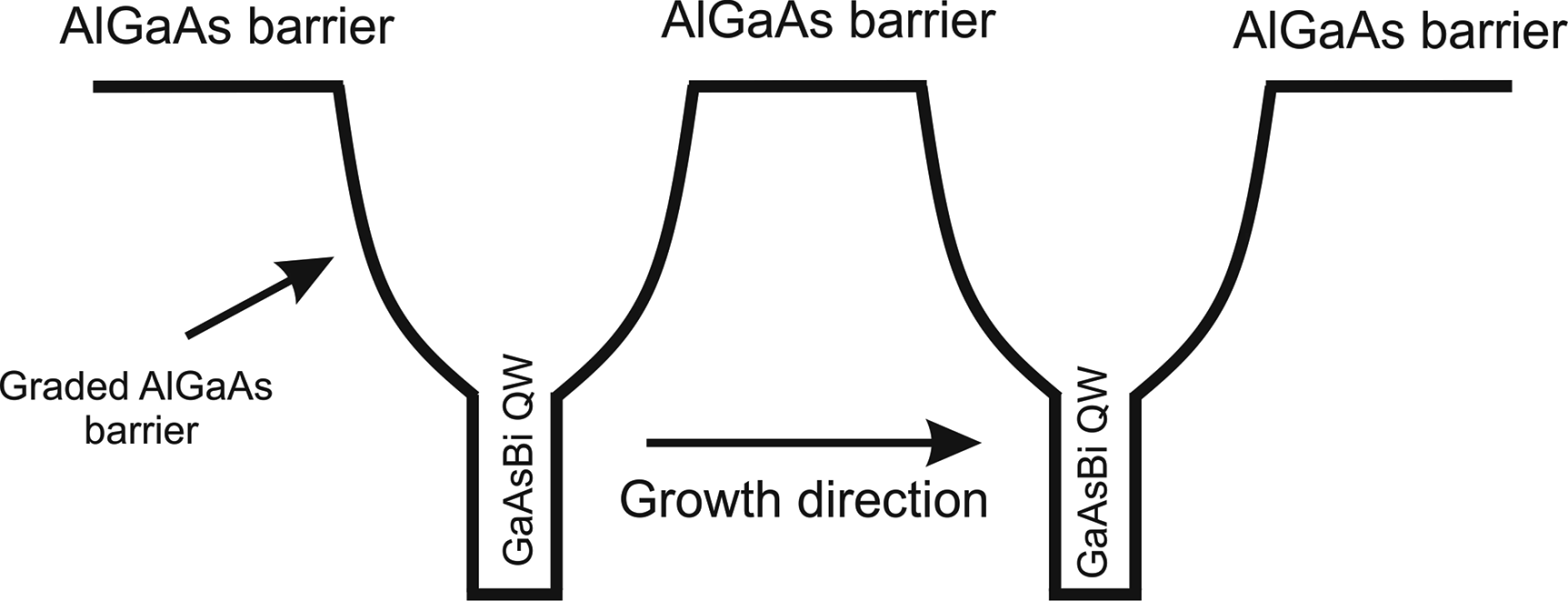
Nominal design of the investigated structure, consisting of two GaAsBi QWs with parabolic graded barriers.

The first AlGaAs-grading barrier is grown at the same temperature changing the Ga–Al ratio from 30 to 0%. The sample growth is then interrupted to decrease the substrate temperature to 420 °C and to reduce the As/Ga ratio flux ratio from 10:1 to 1:1 for a 10 nm-thick GaAsBi quantum well layer. After this stage, fluxes and temperature are restored to As/Ga 10:1 and 665 °C for grading barrier growth. The second QW is grown in the same sequence as the first one, followed by a 100 nm AlGaAs layer and a 5 nm-thick GaAs cap. The nominal structure design of region of interest is presented in Figure 1.

Structural investigations by means of high-resolution TEM measurements of the Bi QDs formed in the GaAsBi layers after annealing were carried out by FEI Tecnai G2 F20 X-TWIN TEM operating at 200 kV, equipped with an EDX detector for elemental mapping and a high-angle annular dark-field (HAADF) detector for *Z*-contrast imaging operating in STEM mode. A 50 μm C2 aperture, corresponding to a probe convergence angle of 9.33 mrad, and a camera length of 150 mm, corresponding to inner collection angle of 41 mrad, were used. The *in situ* TEM investigations were performed with a JEOL 2100F field emission microscope operating at an accelerating voltage of 200 kV. A double-tilt DENS Solutions Lightning D9+ holder was used in combination with commercial Wildfire MEMS chips for temperature control during the *in situ* TEM experiments.

While for ex-situ annealed samples a focused ion-beam FIB preparation route was used, cross-sectional specimens for *in situ* TEM investigations were then prepared using a hybrid approach.^25^ The sample is first conventionally prepared by mechanical grinding, dimpling, and Ar+ ion milling. Afterward, an FIB device is utilized to cut out a thin part of the specimen and transfer it to a MEMS in situ heating chip. This approach minimizes lamellar surface damage as the FIB beam is used for cutting only, but not for FIB polishing.

The *in situ* heating experiments were conducted by ramping up the temperature from RT to 650 °C with a ramping rate of 10 °C/min with holding intervals at 400, 500, 550, and 650 °C for more detailed analysis. Afterward, the sample was cooled to RT with a cooling rate of 10 °C/min. Micrographs were continuously recorded in a bright-field scanning mode at 1024 × 2048 resolution by using Gatan’s DigiScan STEM module and Digital Micrograph software, and the frame interval time was 17.3s.

Sample thickness was determined by two independent methods, modified contamination line method (boundaries between buffer layers were used as a reference) and converged beam electron diffraction pattern analysis. Both methods provided the same sample thickness of 100 nm within 10%.

The crystalline structure was investigated using a high-resolution Rigaku SmartLab X-ray diffractometer.

**Figure 2:**
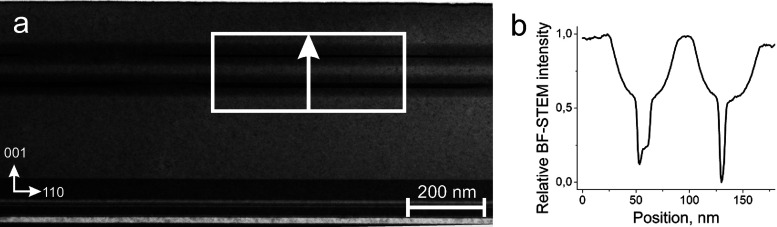
BF-STEM micrograph of the sample before annealing (a) and line scan intensity graph along a white arrow indicating a parabola-shaped QW structure (b).

The cross section of the initial structure of the as-grown sample is investigated by BF-STEM, as depicted in Figure 2. In this regard, the lower part of Figure 2a shows a GaAs wafer along the ⟨110⟩ zone axis, the parabolic GaAs-AlGaAs quantum well, while a relatively dark area corresponds to the GaAsBi layer. Also, the BF-STEM intensity profile in the lower QW area indicates that the Bi distribution is not uniform. This would explain the final distribution of the Bi QD structure after heating. Although growth parameters for both QWs were identical, we find that the first QW is thicker and less uniform than the second one, thus causing bending of the upper structure. Straight interfaces between GaAs and AlGaAs (bright lines in the very bottom of the micrograph) further support this behavior as growth peculiarity. This growth peculiarity in both in-plane and growth directions has been previously observed by various researchers,^10,26^ which is likely to be caused by highly induced strain by incorporating Bi into the lattice or Bi wetting layer nonuniformity.

An inhomogeneous Bi distribution was further confirmed by g002 dark-field (DF) TEM micrographs, which is highly sensitive to variations in the chemistry of the alloy in semiconductors with a zinc blende structure.^11^

**Figure 3:**
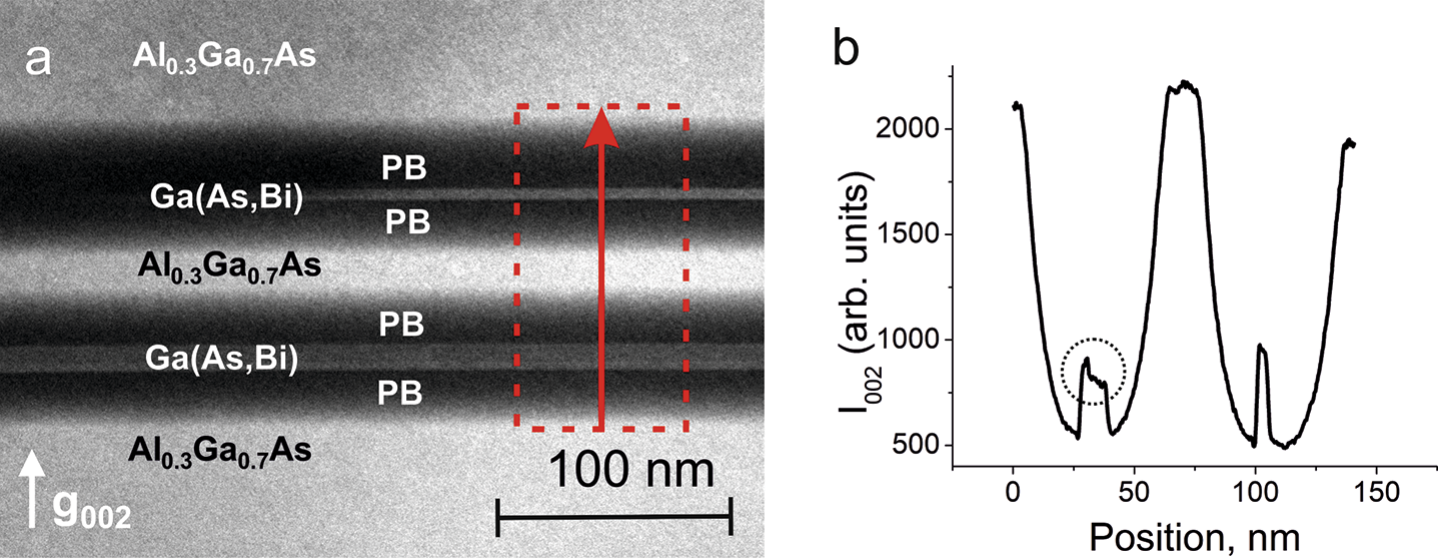
(a) g002 dark-field TEM of the as-grown sample and (b) intensity profiles of the selected area. The area of the nonuniform distribution of Bi in the bottom QW is highlighted.

The g002 DFTEM micrograph presented in Figure 3a indicates that the Bi distribution in the bottom QW is nonuniform and the Bi content is higher at the interfaces. Moreover, the Bi content is higher at the bottom interface than at the top interface (Figure 3b). No similar features were observed on the top QW, despite identical growth parameters.

**Figure 4:**
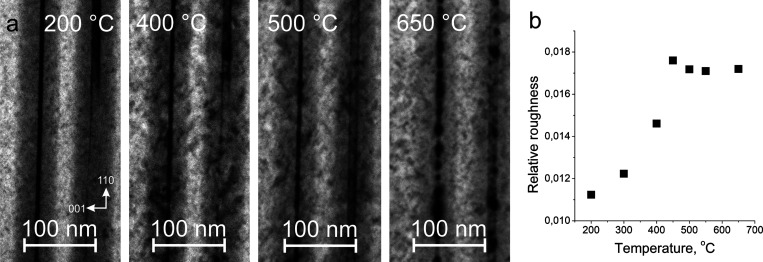
(a) Sample evolution during the heating experiment and (b) sample roughness dependence on heating temperature.

In order to initiate formation of nanometer-sized Bi particles, the sample is heated in the TEM from RT to 650 °C with a ramping rate of 10 °C/min with holding intervals at 400, 500, 550, and 650 °C for more detailed analysis. A heating video, registered at 550 °C, is available in Movie S1. Figure 4 summarizes a sequence of BF-STEM micrographs, taken at different temperatures, as well as sample roughness dependence.

At the beginning, there is no visible change up until 300 °C, where the sample starts to deteriorate, evident in all regions of the sample (GaAs layer, GaAs-AlGaAs parabolic barrier) by the increasing roughness of subsequent sample areas. Roughness was measured for the same area in AlGaAs (50 × 400 nm) by dividing the standard deviation of signal intensity by the average intensity value, using a procedure as described in ref (27) (Figure 4b).

At around 400 °C, formation of Bi QDs starts in the top GaAsBi quantum well, while in the bottom GaAsBi QW, Bi QD formation is not registered up to 450 °C. These initial nanoparticles are round and are limited by the QW thickness. Their size is less than 10 nm, and they show a weak contrast in BF-TEM, mainly due to the thickness of the whole lamella.

**Figure 5:**
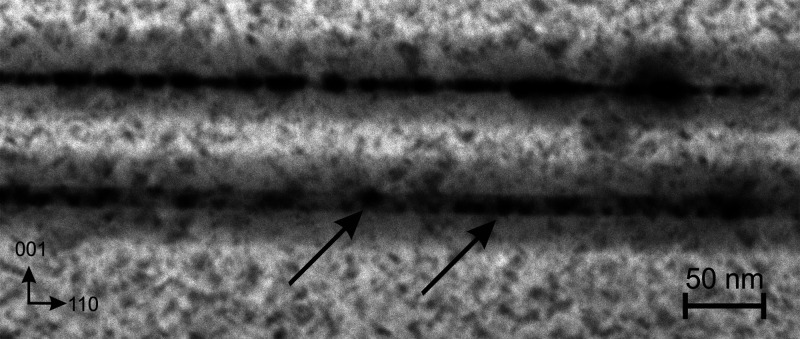
The BF-STEM sample micrograph, taken at 450 °C, illustrates different modes of Bi QD’s formation in the bottom and top QWs, respectively. Arrows highlight Bi QDs, which are attached to interfaces of the bottom GaAsBi QW.

Ramping up the temperature further, more transformations occur at around 450 °C. First, Bi QDs in the top QW start to overgrow the boundaries of QW, and these NPs start to agglomerate by moving in the lateral direction (Figure 5). Second, formation of Bi QDs begins in the bottom QW, but in this case, Bi QD’s formation primarily occurs at the interfaces of the bottom QW (arrowed for clarity).

Further increase of the temperature promotes agglomeration of Bi QDs, thus reducing their number. During the coalescence process, mass transport occurs in the lateral direction, which visualizes as the QDs move in the lateral direction. It is worth noting that, in the bottom QW, while some Bi QDs occupy the whole area of GaAsBi QW, most of them are still attached to the interfaces. Their movement along the lateral direction is also mainly along the interfaces of the QW, or by hopping in the diagonal direction, suggesting a possible mass transport mechanism along the ⟨111⟩ direction, also evident in ref (19). The tendency to form Bi QDs at the interfaces of the QW could be explained by an uneven Bi distribution in the QW, which is evident from the ambient temperature and g002 DF micrograph intensity profiles across the GaAsBi area.

After annealing, the sample was cooled from 650 °C to RT at a rate of 10 °C/min. The sample remained relatively stable, with only minor Bi QD rearrangement that occurred at temperatures higher than 550 °C. This would indicate that the sample was already at a near-equilibrium state at the end of the annealing process. Note that during the heating process, rearrangement of Bi QDs was visible even at lower temperatures. Also, there was no further increase in sample roughness due to possible As loss. A video recorded during the complete cooling process is available as Movie S2.

After *in situ* annealing at the TEM, there are several features visible. First of all, the general uniformity of the sample is much lower. This can be attributed to loss of As during annealing, as annealing was performed in high vacuum conditions. Second, although the total thickness of the QW structure remains unchanged, the degree of parabolicity of the AlGaAs barrier is much lower. Finally, during the annealing process, nanometer-sized Bi particles develop inside the GaAsBi layers and the correlation between the size of the QDs and the width of the GaAsBi QW can be traced in the images, whereas Bi QDs in the top QW tend to be of round shape and the ones in the bottom QW look faceted and tend to be located at the interfaces QW/barrier. In our previous study,^19^ our group has demonstrated that this growth route enables precise control of the diameter of the formed Bi QDs.

Although the detailed mechanism of sample transformation is yet to be determined, it is evident that the mechanisms governing sample transformation of the lamella are different from those for wafer sample transformation.

First of all, the topmost AlGaAs barrier and GaAs cap layers were grown at 665 °C temperature, while transformation on the lamella was registered at a much lower temperature, as low as 300 °C. Second, the general uniformity of the lamella sample is much lower, and there are a lot of defects in both AlGaAs and GaAsBi regions. This can be attributed to an intensive loss of As during annealing, as annealing in the TEM was performed under high vacuum conditions without any extra As supply. In our previous study,^19^*ex-situ* annealing was performed with a GaAs wafer on top and such degradation of the structure was not observed, while As sublimation and formation of defects are widely known^28−30^ for annealing of uncovered GaAs. In this case, annealing temperatures of 700–750 °C were necessary for Bi QD formation, and thus GaAsBi.

Finally, the Bi QD drift velocity of ∼0.5 nm/s (determined by comparing the position of individual Bi QD in adjacent STEM micrographs) is too high for a bulk diffusion process inside the lattice and is comparable with reaction propagation^31^ or amorphization rate^32^ in other systems due to electron beam-induced heating, or Ga droplet movement during GaAs annealing,^33^ thus indicating a different mechanism of Bi QD formation compared to the bulk sample. Annealing the bulk GaAsBi sample, formation of Bi QDs is related to the presence of point defects and Bi atom diffusion toward Bi-rich clusters.^7^ In case of *in situ* annealing of thin lamellas (∼100 nm thickness), it indicates that the mechanism is likely related to the formation of liquid Bi droplets on the surface of lamella^23,24,34^ or other surface-related phenomenon. However, due to different *ex-situ* (wafer) and *in situ* (lamella) annealing conditions (including absence of As during annealing, lamella being extremely thin), this experiment provides valuable information on sample transformation, with the most important being lack of voids and dislocations in the areas of the formed Bi QDs. A planned study of annealing partially cut lamella (prethinned, but before lift-out) next to a bulk wafer would allow to distinguish As out-diffusion and lamella thickness effects.

In summary, in situ TEM was successfully applied to directly observe the formation of Bi quantum dots in the GaAsBi/AlGaAs structure, grown by molecular beam epitaxy. It is shown that lamellar sample transformation occurs in much lower temperatures than in the bulk sample; moreover, a high Bi QD diffusion rate suggests a different quantum dot formation mechanism than in wafer sample annealing. An obvious correlation between the size of the QDs and the width of the GaAsBi QW can be traced from the BF-STEM micrographs, providing a technological route of formation of Bi quantum dot arrays of required QD size.

## Supporting Information Available

Assembly of BF-STEM micrographs, registered during *in situ* heating at 550 °C (AVI)
Complete assembly of BF-STEM micrographs, registered during *in situ* cooling (AVI)

## Supplementary Material

ao4c10631_si_001.avi

ao4c10631_si_002.avi

## Abbreviations

BF-STEM: bright-field scanning transmission electron microscopy
QW: quantum well
PCB: parabolic quantum barrier
